# Social determinants of health and hypertension in women compared with men in the United States: An analysis of the NHANES study

**DOI:** 10.1002/clc.24079

**Published:** 2023-07-10

**Authors:** Li Wang, Hao Zhang, Hong Yao, Chunlin Gong, Jiaoyue Zhong, Dongxue Liu, Zhaoguang Liang

**Affiliations:** ^1^ Department of Cardiology The First Affiliated Hospital of Harbin Medical University Harbin China; ^2^ Department of Clinical Medicine North China University of Science and Technology Tangshan China

**Keywords:** education, employment, ethnicity, gender, hypertension, income, marriage, social determinants

## Abstract

**Background:**

Social determinants of health (SDH) reflecting social deprivation have been developed for population health management. There is a paucity of data on the prevalence of SDH and its associations with prevalent hypertension in women compared with men.

**Methods:**

A total of 49 791 participants aged over 20 years from the 1999–2018 National Health and Nutrition Examination Surveys, were included. Information on the SDH, including race/ethnicity, education level, family income, housing, marriage status, employment, were collected. We calculated the prevalence ratio (PR) for each adverse SDH with prevalent hypertension and uncontrolled hypertension by using Cox regression with equal times of follow‐up assigned to all individuals with adjustment for age, diabetes, taking lipid‐lowering medication, and health behaviors. The population‐attributable fractions (PAF) of the SDH were also assessed.

**Results:**

A lower proportion of low education attainment was observed in women than men (women: 16.8% vs. men: 17.9%, *p* = .003), but women had higher proportions of low family income (15.3% vs. 12.5%, *p* < .001), unmarried state (47.3% vs. 40.9%, *p* < .001), and unemployment (22.7% vs. 10.7%, *p* < .001). All the SDH was significantly associated with hypertension in women. There were significant dose–response associations between the numbers of adverse SDH with hypertension. The total PAF of SDH for prevalent hypertension was greater in women (22.2%) than in men (13.9%).

**Conclusions:**

The widely influential SDH is associated with prevalent hypertension and uncontrolled hypertension. To improve hypertension management, health resources should prioritize socioeconomically disadvantaged groups considering gender differences.

## Graphical abstract

The distributions of social determinants of health (SDH) are different between women and men. There are similar associations between SDH with prevalent hypertension in women and men, as well as uncontrolled hypertension. The population‐attributable fractions of SDH for hypertension were higher in women than men.

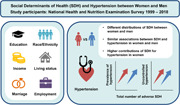

## 1 INTRODUCTION

During the recent three decades, the prevalence of elevated systolic blood pressure (SBP ≥ 140 mmHg) substantially increased, and disability‐adjusted life‐years and deaths associated with elevated blood pressure also increased.^1^ In the United States, the prevalence of hypertension (blood pressure ≥130/80 or taking medication to lower blood pressure) decreased from 47.0% in 1999–2000 to 41.7% in 2013–2014 and then increased to 45.4% in 2017–2018.^2^ Hypertension is a well‐known risk factor for cardiovascular disease (CVD),^3^ lowering blood pressure has been shown to decrease the incidences of stroke, heart attack, and heart failure.

Social determinants of health (SDH), including low socioeconomic status, low education, ethnic differences, suboptimal built environment, and social support networks are increasingly being discussed due to their associations with major diseases.^4^, ^5^ For example, low socioeconomic status, based on household income, education, and employment status, was associated with hazard ratios of 2.3 for CVD mortality and 1.7 for CVD incidence in the UK Biobank cohort.^6^ The individual's social characteristics, including their environment and living conditions, may differ between women and men and performed different effects on developing hypertension. For instance, increased life stressors, work‐related anxiety, and depression, typically have a more significant impact on women with hypertension than on men.^7^ The other SDH, such as marital status and social support, also have different magnitudes on hypertension among women and men.^7^ The PURE study found that there was no gender difference in the association between low education level with incident CVD, the contribution to CVD death by low education was also similar in women and men.^8^ However, depression was more strongly associated with the risk of CVD in men than in women.^8^

Population‐attributable fractions (PAF) represent the percentage of the disease prevalence or incidence in the population that will be prevented by removing a specific risk factor.^9^ Given a public health perspective, the PAF helps to prioritize health budgets and the distribution of resources depending on the proportion of outcomes attributed to a particular exposure. A large number of studies consistently found that a substantial proportion of incident CVD was attributable to hypertension.^10^ The purpose of the present study was to determine the associations and PAFs of hypertension due to SDH by gender among US adults from 1999 to 2000 through 2017–2018. To accomplish these goals, data from 10 cycles of the US National Health and Nutrition Examination Survey (NHANES) were analyzed.

## 2 MATERIALS AND METHODS

### 2.1 Study participants

The NHANES comprises a series of cross‐sectional, national, stratified, multistage probability surveys of the civilian, noninstitutionalized US population. NHANES was designed to assess the health and nutritional status of the US general population. Details of the study design, protocols of data collection, and data sets are publicly available (http://www.cdc.gov/nchs/nhanes.htm). Every participant completed a household interview and underwent a physical examination. From 1999 to 2000, the survey had been conducted in 2‐year cycles. In the present study, 10 cycles conducted from 1999–2000 through 2017–2018 were used. The study protocols were approved by the institutional review board of the National Center for Health Statistics, and written informed consent was obtained from each participant.

### 2.2 Data collection

Participants in the study underwent in‐home interviews, as well as visits to a mobile examination center, where they responded to additional questionnaires and underwent physical examinations and blood sample collection. During the in‐home interview, personal medical history and medication use for diabetes, hypertension, and other conditions were evaluated. Current smoking was defined as having smoked at least 100 cigarettes in life and smoking at present. Current alcohol drinking was defined as taking at least 12 times drinks of any type of alcoholic beverage in the last 12 months. Physical activity was estimated using the form of the Global Physical Activity Questionnaire by asking questions on the intensity, duration, and frequency of physical activity. A different type of physical activity assessment tool was used before 2005–2006. Total metabolic equivalent minutes per week were calculated as the measurement of the physical activity level for the subjects. A higher level of physical activity was defined as having a higher metabolic equivalent/week than the median levels of the metabolic equivalent/week by survey and wave. The number of hours of sleep duration was collected by using a questionnaire. All NHANES examinees were eligible for two 24‐h dietary recall interviews. The first dietary recall interview was collected in‐person in the Mobile Examination Center and the second interview was collected by telephone 3–10 days later. The sodium intake values were calculated based on answers provided by respondents on salt use in cooking or preparing foods in the household (www.ars.usda.gov/ba/bhnrc/fsrg).

Information on age, gender, race/ethnicity, education level, family income, housing, marriage status, employment, and medical history were gathered using a standard questionnaire. Low education attainment was defined as attaining less than a high school education. The income‐to‐poverty ratio (annual family income divided by the poverty threshold adjusted for family size and inflation) was used as a measure of family income.^11^ Low family income was defined as less than 100% of the income‐to‐poverty ratio.^11^ For investigating housing status, the participants were asked “Is this home owned, being bought, rented, or occupied by some other arrangement by you or someone else in your family?” Employed status was dichotomized as unemployed and employed, student, or retired (https://wwwn.cdc.gov/nchs/nhanes/analyticguidelines.aspx).

Details on the data collection are described on the website (https://wwwn.cdc.gov/nchs/nhanes/analyticguidelines.aspx). Weight and height were measured during the physical examination, and body mass index (BMI) was calculated as weight in kilograms divided by height in meters squared.^12^ The participants were asked to rest quietly in a seated position for at least 5 min, then trained staff used a mercury sphygmomanometer to measure blood pressure. Three blood pressure measurements were recorded and the mean of all measurements was used in analyses. Hypertension was defined as SBP of 130 mmHg or higher, diastolic blood pressure (DBP) of 80 mmHg or higher, or currently taking medication to lower high blood pressure.^13^ Before 2017, uncontrolled hypertension referred to a condition in which, despite taking antihypertensive medication, SBP ≥ 140 mmHg or DBP ≥ 90 mmHg.^14^ In 2017, the American College of Cardiology/American Heart Association in partnership with other professional societies published a blood pressure guideline that redefined hypertension as a persistent average SBP ≥ 130 mm Hg or DBP ≥ 80 mm Hg, and reduced the SBP/DBP goal of therapy to <130/80 mm Hg.^15^ Therefore, for the survey conducted during 2017–2018, uncontrolled hypertension was defined as SBP ≥ 130 mmHg or DBP ≥ 80 mmHg under medical therapy.

### 2.3 Statistical analysis

The appropriate weights and design factors were invoked in all the analyses to account for the multistage probability sampling design of the survey. Demographic and other characteristics of study participants were described in means (95% confidence intervals [CI]) for continuous variables and percentages (95% CIs) for categorical variables. The percentages in different groups were compared using *χ* ^2^ tests. As suggested by previous studies,^16^ we used Cox regression with a constant for the time variable assigned to all individuals and with robust variance estimates to assess the association between the variables of SDH with prevalent hypertension and uncontrolled hypertension by calculating the prevalence ratio (PR) with adjustment for covariates. We selected a priori potential confounders for adjustment in multivariable models, including age, current smoking (yes/no), current drinking (yes/no), high level of physical activity (yes/no), sleep duration, sodium intake, BMI, diagnosed diabetes (yes/no), and taking lipid‐lowering medications (yes/no).

We calculated the population‐level risk attributable for the six risk factors of SDH, including low education attainment, not Non‐Hispanic White, low family income, not homeowner, unmarried state, and unemployment using the approach described by Eide and Gefeller ^17^ and the averisk R package developed by Ferguson and colleagues.^18^ PAFs and associated 95% CIs quantified the proportional reduction in disease prevalence that would be achieved if the risk factors were theoretically removed from the population. PAFs were calculated with adjustment for the aforementioned covariates.

## 3 RESULTS

The present study was limited to participants aged 20 years or older (*n* = 55 081). In addition, those who were pregnant or lactating at the time of examination or with unknown pregnancy status (*n* = 2639) or did not have hypertension information (*n* = 2651) were excluded. After exclusion, a total of 49 791 participants were included in the final analysis sample (Table 1).

**Table 1:** The characteristics of subjects by gender.

	Women (*n* = 24 864)	Men (*n* = 24 927)	*P* value
Age, year	48.5 (48.1–48.9)	46.3 (45.9–46.7)	<.001
Education (%)			<.001
Less than 9th grade	5.7 (5.3–6.1)	6.3 (5.8–6.7)	
9–11th grade (Includes 12th grade with no diploma)	11.1 (10.4–11.8)	11.7 (10.9–12.4)	
High school graduate/GED or equivalent	23.5 (22.7–24.3)	24.9 (23.8–25.9)	
Some college or AA degree	32.6 (31.7–33.6)	29.0 (28.2–29.8)	
College graduate or above	27.1 (25.7–28.5)	28.2 (26.7–29.8)	
Race (%)			<.001
Non‐Hispanic White	68.6 (66.5–70.7)	68.7 (66.7–70.7)	
Non‐Hispanic Black	11.8 (10.5–13.0)	10.4 (9.4–11.4)	
Non‐Hispanic Asian^a^	5.6 (4.6–6.5)	5.2 (4.3–6.2)	
Other Hispanic	5.7 (4.8–6.6)	5.4 (4.5–6.2)	
Mexican American	7.2 (6.2–8.2)	8.7 (7.6–9.8)	
Other Race—Including multiracial	6.8 (6.1–7.4)	6.8 (6.2–7.5)	
Family income‐to‐poverty ratio	2.9 (2.8–3.0)	3.1 (3.0–3.1)	<.001
Living status (%)			.414
Owned or being bought	68.5 (67.1–69.9)	67.8 (66.4–69.2)	
Rented	29.5 (28.2–30.9)	30.3 (28.8–31.6)	
Other arrangement	2.0 (1.7–2.2)	1.9 (1.7–2.2)	
Marriage status (%)			<.001
Married	52.7 (51.6–53.8)	59.1 (57.9–60.2)	
Widowed	9.9 (9.4–10.4)	2.4 (2.2–2.6)	
Divorced	12.0 (11.4–12.5)	8.2 (7.7–8.7)	
Separated	2.9 (2.7–3.2)	2.1 (1.8–2.3)	
Never married	15.8 (14.9–16.7)	20.0 (19.0–21.0)	
Living with partner	6.7 (6.2–7.2)	8.1 (7.6–8.7)	
Body mass index (kg/m^2^)	28.9 (28.7–29.1)	28.6 (28.5–28.8)	<.001
Current smoking (%)	39.7 (38.6–40.7)	53.6 (52.5–54.7)	<.001
Current drinking (%)	63.8 (62.5–65.2)	81.0 (80.0–82.0)	<.001
High level of physical activity (%)	38.1 (36.7–39.5)	46.0 (44.6–47.4)	<.001
Sleep duration (h)	7.2 (7.1–7.2)	7.0 (7.0–7.1)	<.001
Sodium intake (mg)	2981.1 (2956.1–3006.1)	4113.1 (4078.3–4147.9)	<.001
Hypertension (%)	48.4 (47.4–49.3)	53.9 (52.8–55.0)	<.001
Uncontrolled Hypertension (%)^b^	39.5 (38.1–41.0)	37.9 (36.1–39.8)	.235
Diabetes (%)	11.0 (10.5–11.6)	11.8 (11.3–12.3)	.007

^a^ Non‐Hispanic Asian was listed as a separate race and ethnicity since 2011–2012, the proportions of non‐Hispanic Asian were calculated based on the surveys from 2011–2012 to 2017–2018.

^b^ The proportions of uncontrolled hypertension were calculated among the subjects being diagnosed with hypertension and taking antihypertensive medication.

**Figure 1:**
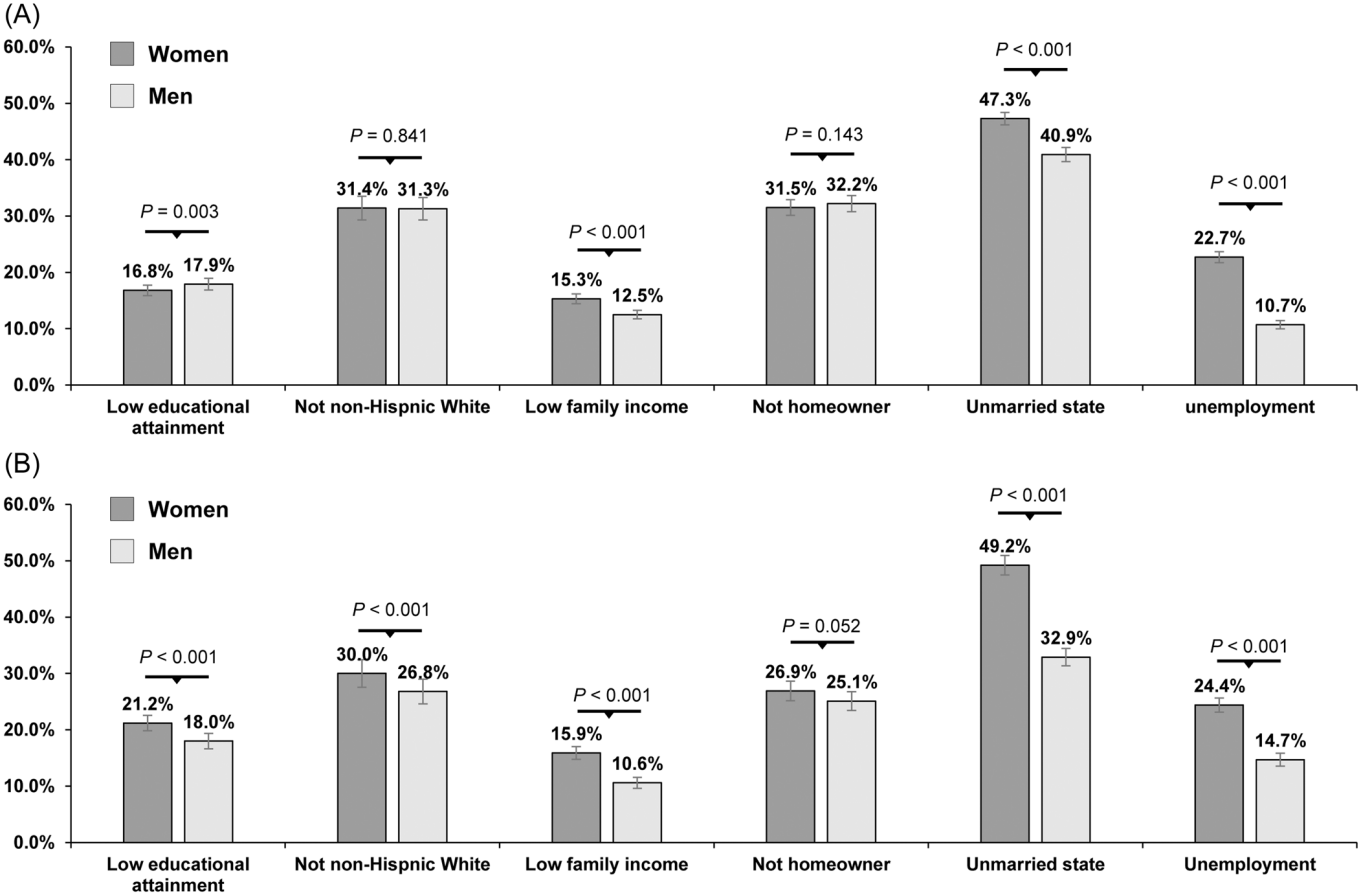
Prevalence of social determinants of health in women and men. (A) All the participants, (B) Participants with treated hypertension.

Men had a higher proportion of lower educational attainment than women (women: 16.8% vs. men: 17.9%, *p* = .003). By contrast, women had significantly higher proportions of low family income (women: 15.3% vs. men: 12.5%, *p* < .001), unmarried state (women: 47.3% vs. men: 40.9%, *p* < .001), and unemployed (women: 22.7% vs. men: 10.7%, *p* < .001) than men (Figure 1A). There were similar proportions of non‐Hispanic White and not homeowner between women and men. Men had a significantly higher prevalence of hypertension than women (women: 48.4% vs. men: 53.9%, *p* < .001). Among the patients with hypertension and taking antihypertensive medication, male patients had significantly lower proportions of low educational attainment, not non‐Hispanic White, low family income, unmarried state, and unemployment than female ones (all *p* < .001) (Figure 1B).

**Figure 2:**
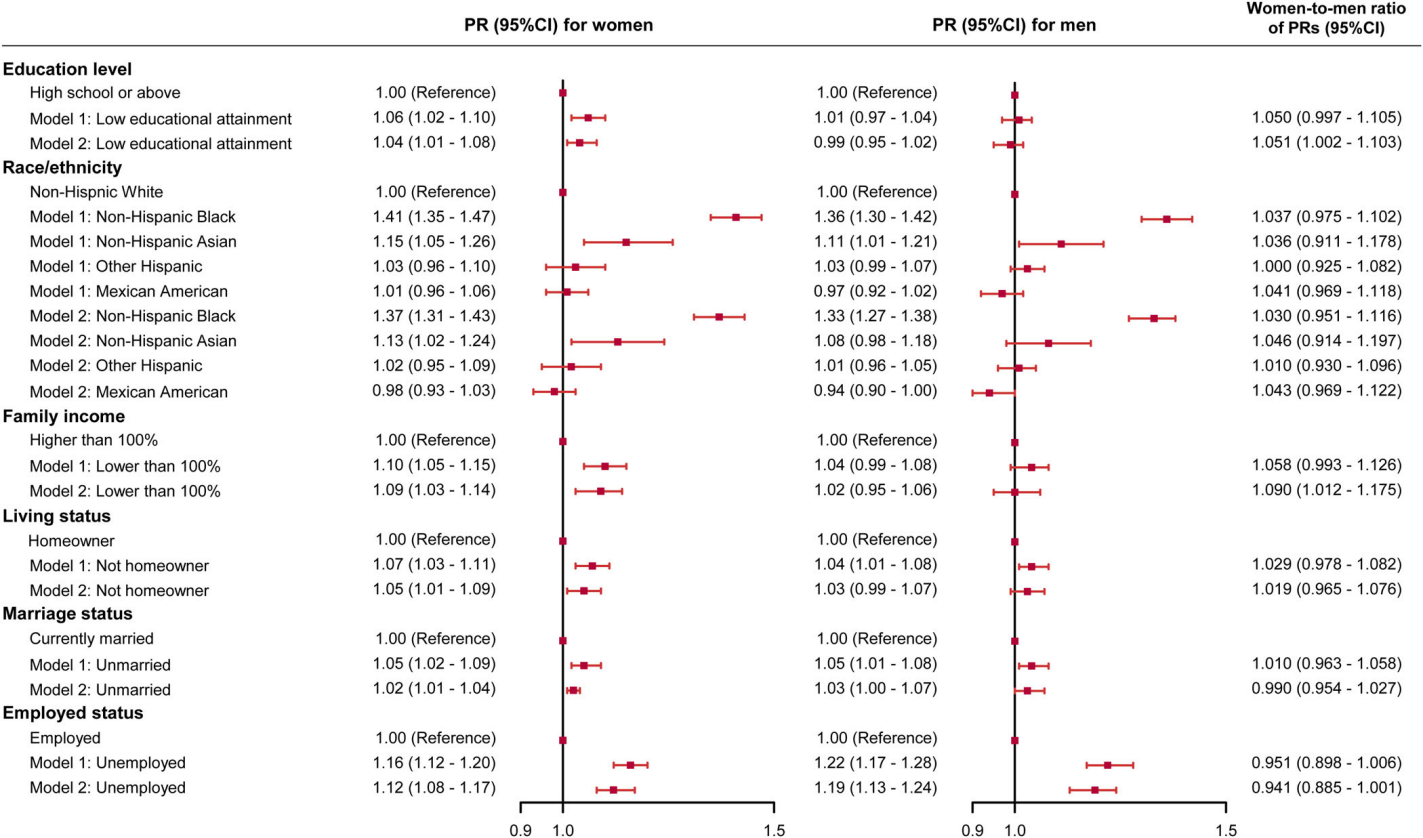
Associations between social determinants of health with hypertension in women and men. Model 1 was adjusted for age, body mass index, diagnosed diabetes (yes/no), and taking lipid‐lowering medications (yes/no); Model 2 was additionally adjusted for current smoking (yes/no), current drinking (yes/no), high level of physical activity (yes/no), sleep duration, and sodium intake. PR, prevalence ratio.

Associations of each SDH with prevalent hypertension in women and men are shown in Figure 2. All the SDH were significantly associated with higher PRs of hypertension in women. Compared with non‐Hispanic White, non‐Hispanic Black (PR = 1.37, 95% CI = 1.31–1.43) and non‐Hispanic Asian (PR = 1.13, 95% CI = 1.02–1.24) had significantly higher PRs of hypertension in women, while only non‐Hispanic Black (PR = 1.33, 95% CI = 1.27–1.38) had significantly higher PR of hypertension in men after adjusting for covariates. Low family income (PR = 1.02, 95% CI = 0.95–1.06), not homeowner (PR = 1.03, 95% CI = 0.99–1.07), and being unmarried (PR = 1.03, 95% CI = 1.00–1.07) were not significantly associated with hypertension in men after adjusting for health behaviors. Unemployment (PR = 1.19, 95% CI = 1.13–1.24) was significantly associated with hypertension in men. Low education attainment and low family income were slightly more strongly associated with hypertension in women than in men, whereas the PRs for other SDH were similar among women and men (Figure 2).

**Figure 3:**
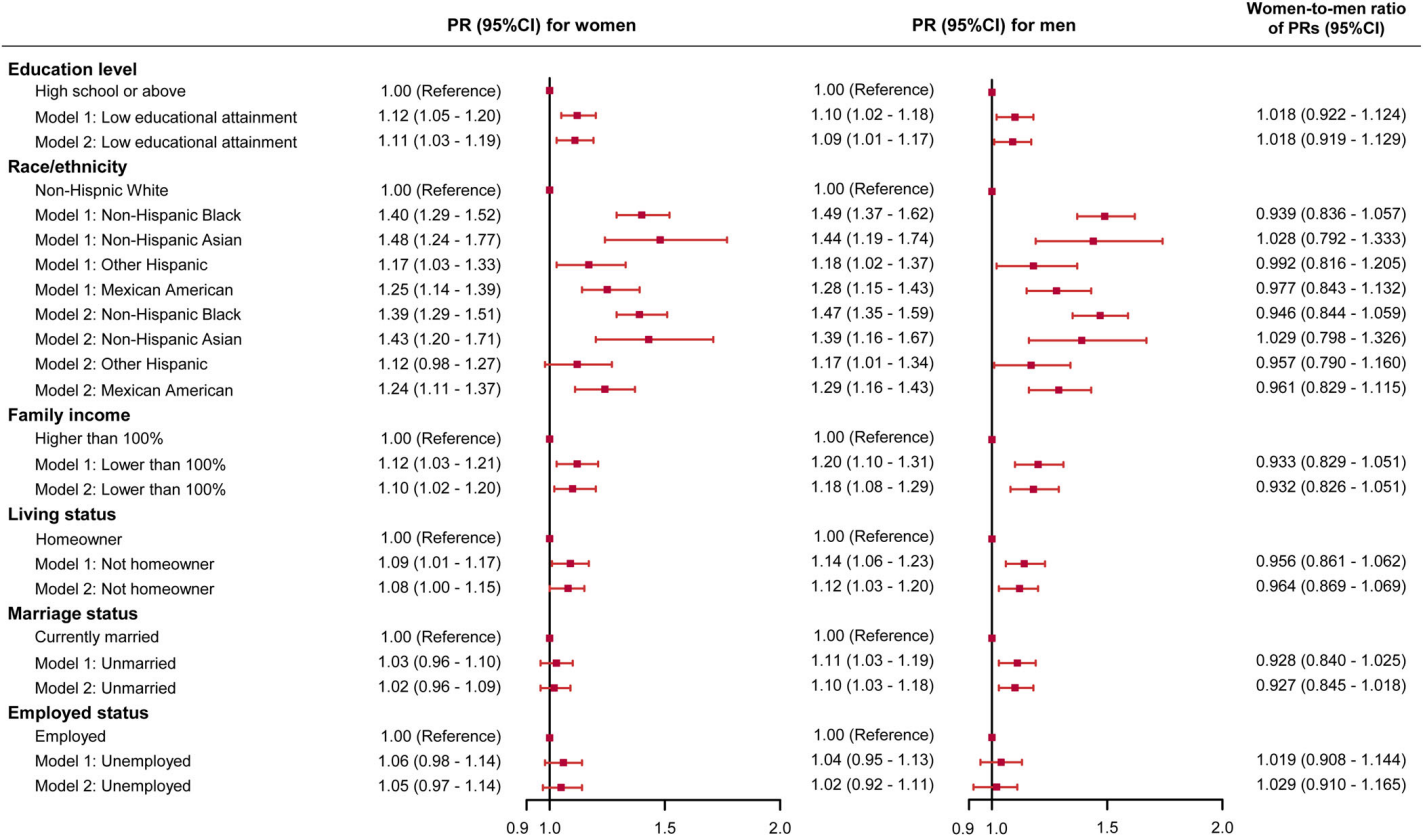
Associations between social determinants of health with uncontrolled hypertension in women and men. Model 1 was adjusted for age, body mass index, diagnosed diabetes (yes/no), and taking lipid‐lowering medications (yes/no); Model 2 was additionally adjusted for current smoking (yes/no), current drinking (yes/no), high level of physical activity (yes/no), sleep duration, and sodium intake. PR, prevalence ratio.

Associations of each SDH with uncontrolled hypertension in women and men were assessed among the participants who had been diagnosed with hypertension and taken antihypertensive therapy (Figure 3). Low education attainment and low family income were significantly associated with uncontrolled hypertension both in women and men. Except for other Hispanic in women, all the other races/ethnicities had significantly higher PRs of uncontrolled hypertension compared with non‐Hispanic White both in women and men. Not homeowner (PR = 1.12, 95% CI = 1.03–1.20) and unmarried (PR = 1.10, 95% CI = 1.03–1.18) were significantly associated with higher PRs of uncontrolled hypertension in men.

**Figure 4:**
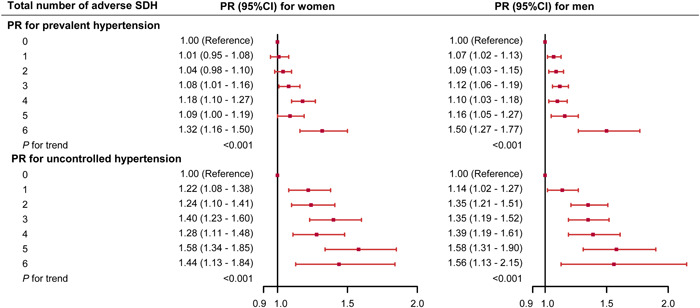
Associations between number of adverse social determinants of health with prevalent hypertension and uncontrolled hypertension in women and men. Adverse social determinants of health include low education attainment, not non‐Hispanic White, low family income, not homeowner, unmarried state, unemployment. Model were adjusted for age, current smoking (yes/no), current drinking (yes/no), high level of physical activity (yes/no), sleep duration, sodium intake, body mass index, diagnosed diabetes (yes/no), and taking lipid‐lowering medications (yes/no). PR, prevalence ratio; SDH, social determinants of health.

Multiple‐adjusted PR for prevalent hypertension and uncontrolled hypertension according to the number of adverse SDH are shown in Figure 4. Overall, there were significant dose–response associations between the numbers of adverse SDH with hypertension. For the participants with all six risk factors, the PRs were increased to 1.32 (95% CI = 1.16–1.50) and 1.50 (95% CI = 1.27–1.77) for women and men, respectively. Similar results were also observed for the associations between the numbers of adverse SDH with uncontrolled hypertension.

Approximately 22.2% of the PAFs for hypertension were attributed to the SDH in women and 13.9% in men (Table 2). Low education attainment, not homeowner, and being unmarried contributed substantially to prevalent hypertension in women and men, the PAFs of these factors were also higher in women than men. Among the participants with hypertension, the PAFs of SDH for uncontrolled hypertension were similar in women (22.6%) and in men (23.2%) (Table 2). Not non‐Hispanic White contributed substantially to the risk of uncontrolled hypertension in women (8.3%) and men (9.2%). Low educational attainment (5.6%) and low family income (4.2%) had similar contributions to the PAFs of uncontrolled hypertension in women, whereas low family income (7.7%) had the second largest contribution in men.

**Table 2:** Population‐attributable fractions (PAFs) and 95% confidence interval (CI) for six social determinants on prevalent hypertension and hypertension not controlled by gender.

	Women	Men
PAF for prevalent hypertension (%)		
Low educational attainment	5.4 (4.4–6.5)	1.9 (1.1–2.9)
Not non‐Hispanic White	1.6 (0.2–3.5)	0.7 (−0.1 to 1.7)
Low family income	0.6 (0.1–1.2)	0.3 (−0.5 to 1.1)
Not homeowner	8.2 (7.0–9.8)	5.6 (4.5–6.8)
Unmarried state	5.7 (4.0–7.6)	3.1 (2.2–4.2)
Unemployment	0.7 (−1.1 to 2.5)	2.3 (1.8–2.9)
PAF for uncontrolled hypertension (%)		
Low educational attainment	5.6 (2.7–8.7)	2.2 (−0.1–4.8)
Not non‐Hispanic White	8.3 (2.5–15.1)	9.2 (5.9–12.6)
Low family income	4.2 (0.5–8.0)	7.7 (0.4–15.2)
Not homeowner	2.1 (0.3–4.1)	1.6 (−0.1 to 3.4)
Unmarried state	0.8 (−6.2 to 7.8)	1.6 (0.2–3.2)
Unemployment	1.6 (−0.7 to 4.1)	0.9 (−2.0 to 3.9)

*Note*: Data are PAFs with 95% CIs in parentheses. All adverse social determinants of health (SDH) are added to the model with adjusted for age, current smoking (yes/no), current drinking (yes/no), high level of physical activity (yes/no), sleep duration, sodium intake, body mass index, diagnosed diabetes (yes/no), and taking lipid‐lowering medications (yes/no).

## 4 DISCUSSION

Using a nationally representative sample of the adult population, our study obtains three major findings. First, women have a more unfavorable SDH than men. This finding was supported by much higher prevalence of low family income, being unmarried, and unemployed in women than in men. Second, despite gender differences in SDH levels, the magnitudes of the associations with prevalent hypertension and uncontrolled hypertension for most SDH were similar in women and men. Third, the contribution of SDH for prevalent hypertension were higher among women than men, especially for low education attainment, not homeowner, and being unmarried. Our results suggest that one‐quarter of hypertensive cases can be substantially avoided with improved SDH for women.

Several studies have reported the impact of SDH on hypertension. Similar to our study, the findings of the Atherosclerosis Risk in Communities Study (ARIC) also found that better individual‐level SDH was associated with lower hypertension incidence in later life.^19^ The association between education and hypertension prevalence has been relatively consistently reported by previous studies, high educational attainment improves the awareness, treatment, and control of hypertension.^20^ Blood pressure in people with high educational attainment might be better controlled than in those with low educational attainment.^21^ For example, a study in South Korea reported an association between educational attainment and better awareness of blood pressure among women.^22^ Pandit et al. found that those who had higher formal education were more aware of their overall health and more compliant with medical therapy, which ultimately lead to better blood pressure control.^23^ Based on a cohort conducted in rural Vietnam, less formal education was associated with a lower likelihood of hypertension in men, but this relationship was completely reversed in women.^24^ People with higher educational levels were likely to have higher access to healthcare services and better performance in disease prevention and management.^25^ Adults with lower education attainment more generally were less likely to initiate and receive preventive treatments.^26^ In our study, we not only confirmed the positive associations between SDH and prevalent hypertension, but also presented the gender differences in the associations and contributions of SDH for hypertension. We found the magnitude of the associations between low educational attainment with prevalent hypertension was slightly higher in women than that in men, the contributions to prevalent hypertension and uncontrolled hypertension of this risk factor were also higher in women. Similar to our findings, the PURE study also found that low education was the largest contributing risk factor for CVD death (PAF, 11.6% in women vs. 10.3% in men).^8^ This finding enforced the understanding of the role of gender‐related factors in the prevalence and control of hypertension. Another study also provide evidence from China that lower socioeconomic status was associated with incident hypertension and women were more susceptible.^27^ Therefore, programs to reduce hypertension prevalence and improve hypertension control should be given high priority among women with low educational attainment. On the other hand, strengthening education attainment (or addressing health barriers in low‐education groups) was important to improve hypertension management.

Marital status has also been shown to play an important role in blood pressure. Unmarried men were reported to be nearly 50% more likely to have hypertension compared to married men, while, in women, being widowed increased the risk of hypertension by 92%.^28^ There is little literature that investigates the role of employment as well as other gender roles in association with hypertension. Our study found that unemployment was consistently significantly associated with prevalent hypertension but not hypertension control in both women and men, however, the contribution of unemployment to prevalent hypertension was higher in men than that in women. The cause of these relationships is still unclear, but it may be secondary to lifestyle and cultural differences. For instance, calorie‐heavy foods are typically inexpensive and rapid to prepare and consume in high‐income countries. Hence, while those with lower income or unemployed in a resource‐rich country tend to eat unhealthier fast food and processed sugars, leading to high blood pressure.^7^

Several mechanisms may explain our findings of SDH relating to hypertension. There are substantial previous literatures concerning the etiology of hypertension specifically focusing on modifiable risk factors related to diet, inactivity, tobacco and alcohol consumption, and obesity.^29^ Social factors, particularly individual socioeconomic status, may affect the prevalence/incidence of hypertension via these behavioral factors.^30^ It is well known that education, income, and gender inequality can influence life decisions and resource allocation. People with low socioeconomic status may have limited access to social and economic resources, recreational facilities, and healthy foods, which may directly or indirectly affect individuals’ ability to engage in healthy behaviors. In a multicohort study, Wang et al. found that low socioeconomic status was significantly associated with an increase of four times in the odds of initiating physical inactivity, an increase of more than two times in the odds of continuing physical inactivity and of continuing smoking.^30^

This study had several limitations. First, NHANES comprised a series of cross‐sectional surveys, so longitudinal changes in SDH and blood pressure at an individual level could not be evaluated. Second, many important SDH, such as living environment and regional economic level and medical resources, were not measured and could not be included in this analysis. Third, although most risk factors were measured using validated methods, measurement error was possible, especially when data were self‐reported.

In conclusion, the widely influential SDH is significantly associated with hypertension and hypertension control. These findings support the need for urgent actions and reinforced efforts to address socioeconomic inequalities in hypertension management. To improve hypertension management, more health resources should prioritize socioeconomically disadvantaged groups with considering gender differences when designing and implementing secondary prevention programs.

## CONFLICT OF INTEREST STATEMENT

The authors declare no conflict of interest.

## Data Availability

The data sets generated and analyzed during the current study are available in the NHANES repository (https://wwwn.cdc.gov/nchs/nhanes/Default.aspx).
